# Initiation of the Hypothalamic–Pituitary–Gonadal Axis in Young Girls Undergoing Central Precocious Puberty Exerts Remodeling Effects on the Prefrontal Cortex

**DOI:** 10.3389/fpsyt.2019.00332

**Published:** 2019-05-10

**Authors:** Di Yang, Wenjing Zhang, Yaxin Zhu, Peining Liu, Bo Tao, Yuchuan Fu, Yu Chen, Lu Zhou, Lu Liu, Xin Gao, Xiaozheng Liu, Leah H. Rubin, John A. Sweeney, Zhihan Yan

**Affiliations:** ^1^Department of Radiology, the Second Affiliated Hospital and Yuying Children’s Hospital of Wenzhou Medical University, Wenzhou, China; ^2^Department of Radiology, Zhejiang Hospital, Hangzhou, China; ^3^Department of Radiology, the Center for Medical Imaging, West China Hospital of Sichuan University, Chengdu, China; ^4^Department of Child Health Care, the Second Affiliated Hospital and Yuying Children’s Hospital of Wenzhou Medical University, Wenzhou, China; ^5^Department of Radiology, People’s Hospital of Deyang City, Deyang, China; ^6^Department of Psychiatry, University of Illinois at Chicago, Chicago, IL, United States; ^7^Department of Psychiatry and Behavioral Neuroscience, University of Cincinnati, Cincinnati, OH, United States

**Keywords:** central precocious puberty, hypothalamic-pituitary-gonadal axis, cortical thickness, estradiol, psychological development

## Abstract

Central precocious puberty (CPP) has been shown to exert significant effects on psychosocial development. These early puberty-related hormones and psychosocial functional changes are considered to be associated with specific brain development. However, the biological mechanisms underlying the sculpting of human brain architecture and modulation of psychosocial transformation by puberty-related hormonal maturation remain elusive, especially during the early phase of CPP. The current investigation aims to specify the brain regions in which early hormone-related maturation effects occur during CPP and their relationships with psychological functions. 65 young girls (aged 4.3–8.0 years) underwent structural imaging on a 3T MR system, completed psychological tests and performed the gonadotropin-releasing hormone (GnRH) stimulation test to identify hormonal manifestations of hypothalamic–pituitary–gonadal axis (HPG axis) activation. Based on the GnRH test, 28 young girls were identified with CPP, whereas the other 37 girls were identified with non-central precocious puberty (NCPP). Cortical parameters were calculated and compared between the two groups after adjusting for age, weight, and height. Brain regions showing group differences were extracted and correlated with serum hormone levels and psychological parameters. The CPP girls showed thinner cortices primarily in the right rostral middle frontal cortex. This morphological difference was positively correlated with stimulated estradiol (E2) levels. Further, higher E2 levels were significantly associated with higher hyperactivity scores. Premature HPG axis activation in CPP girls at an early stage appears to exert remodeling effects on brain anatomy, primarily in the prefrontal cortex, which may affect psychological development following the emergence of robust changes in sex hormones.

## Introduction

Precocious puberty (PP) is an important issue that affects approximately 1 in 5,000–10,000 children (1, 2). In addition to physical problems, PP has been reported to be related to higher rates of psychological problems in patients when compared to healthy children (3, 4). A previous longitudinal study of 2,607 girls with early menarche showed that early puberty might increase the risk of behavioral problems in young adolescent girls. Early maturing girls are at risk of persistently higher delinquency and stronger negative peer influences (5). These cognitive, behavioral, and emotional functioning changes are thought to be associated with specific brain development (6–8). However, few studies have specified the brain regions where particular maturational effects occur during this sensitive period within this special group. Identifying brain regions that contribute to psychological functioning in PP girls may be critical for interventions to prevent later negative outcomes.

From an etiological perspective, PP may be subdivided into gonadotropin-releasing hormone (GnRH)-dependent causes, which are often called central precocious puberty (CPP), and GnRH-independent causes [non-central precocious puberty (NCPP)]. The major differences between these causes are the hypothalamic mechanism based on hypothalamic–pituitary–gonadal (HPG) axis activation and corresponding endocrine changes, including robust increases in both gonadotropin and sex hormones. Since hormone-dependent brain organization occurs during normal development in adolescents and variations lead to individual differences in cognitive processes, adult behavior and sex-biased risks (9, 10), specific hormone-related maturational effects on brain morphometry may be related to CPP, which may lead to different cognitive or behavioral characteristics between CPP and NCPP individuals.

Standard treatment for CPP is based on postponement of pubertal development by blockade of the HPG axis with gonadotropin-releasing hormone analogs (GnRHa), leading to abolition of gonadal sex hormone synthesis (11). Although the hormonal and auxological effects of GnRHa are well researched, their influences on the brain are largely unknown. Understanding the mechanism of abnormal activation of the HPG axis and the effects on brain morphometry and cognitive and behavioral development may clarify the mechanisms underlying this therapeutic effect and help develop targeted treatments or preventive measures for undesirable mental changes.

Over the last two decades, some studies have focused on the brain changes of CPP individuals, but the findings have mainly focused on suprasellar arachnoid cysts and hypothalamic hamartomas (12). Most CPP cases in girls do not have a detectable CNS lesion and are described as idiopathic CPP (13). However, idiopathic CPP girls have been found to have an increased pituitary gland height and area (14–16). Recently, more automatic and precise neuroimaging analytical methods, such as cortical thickness, have been widely used in neurophysiological studies of normal neurodevelopment and psychiatric disorders (17–19), which have no obvious histological abnormalities on MRI plain scans. Since the cerebral cortex can be a highly folded outer layer of gray matter tissue that plays a key role in cognitive and behavioral functions (20), alterations of the brain morphometry, which programs a variety of psychological functions during development or disease, can be captured by measuring the cortical thickness across the whole brain.

Under these circumstances, we conducted structural MRI studies of CPP and NCPP girls with the aim of identifying specific HPG axis activation-related influences on brain organization during the initiation of PP and determining the extent to which these early neuroendocrine changes modulated the brain microstructures responsible for the changes (or differences) in cognitive function, behaviors, and emotions in CPP girls. The goal was to establish a model of “neuroendocrine-brain morphology-psychology” and to unveil the influence of early activation of the HPG axis on brain morphometry and cognition, behaviors, and emotions.

## Materials and Methods

### Participants

The study was approved by the local research ethics committee. Written informed consent was obtained from the parents or guardians, and study assent was obtained from the girls to establish their willingness to participate. Sixty-five right-handed girls with PP aged 4.3–8.0 years were recruited *via* the Second Affiliated Hospital of Wenzhou Medical University. The exclusion criteria were as follows: 1) an IQ < 70 estimated by the Chinese Wechsler Intelligence Scale for Children (C-WISC) (21); 2) a history of neurological or psychiatric disorders in the study participants or their first-degree relatives, chronic medical illness, learning disabilities, or use of medicines known to affect hormone levels or central nervous system functioning; 3) born at <37 weeks gestational age; 4) having ever menstruated; 5) a history of a head injury; or 6) contraindications for MRI scanning. Details of the demographics of all subjects are shown in **Table 1**.

**Table 1:** Demographics and clinical characteristics of the CPP and NCPP girls.

Characteristic		CPP (n = 28)		NCPP (n = 37)		*p*
		Mean	SD	Mean	SD	
Age (years)		7.23	0.78	7.02	0.92	0.33
Weight (kg)		26.08	4.49	26.00	4.49	0.94
Height (m)		1.26	0.06	1.24	0.07	0.45
BMI (kg/m^2^)		16.36	2.39	16.60	1.78	0.64
LH (IU/L)	Peak	9.46	8.12	3.22	1.12	0.00*
FSH (IU/L)	Peak	17.13	6.63	14.57	5.35	0.09
E2 (pg/ml)	Peak	36.56	20.51	26.79	13.58	0.02*
TES (ng/ml)		0.22	0.05	0.21	0.05	0.61
PRL (ng/ml)		12.89	8.10	10.90	5.92	0.26
COR (µg/ml)		12.71	6.32	11.35	6.00	0.38
IQ (total)		82.82	14.01	92.05	13.98	0.09
CBCL (total)		7.70	8.00	9.39	9.14	0.45
HAMA (total)		3.47	3.55	3.65	3.00	0.87

CPP, central precocious puberty; NCPP, non-central precocious puberty; SD, standard deviation; BMI, body mass index (calculated as weight/height^2^); LH, luteinizing hormone; FSH, follicle stimulating hormone; E2, estradiol; TES, testosterone; PRL, prolactin; COR, cortisol; IQ, intelligence quotient; CBCL, Child Behavior Checklist; HAMA, Hamilton Anxiety Rating Scale. *p < 0.05.

PP was diagnosed by the chief of the Child Health Care Centre in our hospital based on early breast development in the girl (Tanner stage 2 breast development) before 8 years of age (22, 23). The two subtypes were with versus without HPG axis activation (CPP and NCPP) and were distinguished by the GnRH-stimulated luteinizing hormone (LH) levels through the GnRH stimulation test.

Since it is hard to convince the parents or guardians to let their normally developed girls to complete the invasive blood collection (especially with the injection of medication) and an MRI scan session, we have not got enough normal controls of the same age. Thus, we can only choose to examine these younger girls with PP specifically.

### GnRH Stimulation Test

HPG axis activity increases with the onset of puberty, as evidenced by increasing numbers and amplitude pulses of gonadotropins, LH, and follicle stimulating hormone (FSH). Based on the pulsatile secretion feature, basal gonadotropin measurements poorly discriminate between prepubertal and early pubertal children (23). The GnRH stimulation test, which is also known as the LH releasing hormone (LHRH) stimulation test, uses LHRH (a ten-peptide bioactive substance secreted from the hypothalamus) to stimulate the synthesis and secretion of a large gonadotropin pulse and release LH and FSH, which can be evaluated as the reserve capacity of pituitary gonadotropin cells (24).

The GnRH stimulation test was performed after imaging data acquisition in examiners blinded to the imaging findings. The interval between the tests was less than 1 week. Following overnight fasting, the participants were asked to arrive at the hospital at approximately 8:00 am. LHRH was injected as an intravenous bolus of 2.5 μg/kg (maximum dose < 100 μg) (25) through an indwelling catheter. Four to five milliliters of blood were collected immediately before injection (0-min sample), and then 2 ml were collected at 30 and 60 min after the injection.

The blood samples were sent for analysis to the hospital clinical laboratory (the 0-min sample was delivered immediately, and the 30-min sample was sent with the 60-min sample). There, the samples were centrifuged, separated, and assayed. We assayed the LH, FSH, estradiol (E2), testosterone (TES), prolactin (PRL), and cortisol (COR) concentrations separately. A detailed description of this process is provided in the supporting information (**Supplementary Methods**).

The peak GnRH-stimulated gonadotropin concentrations are low but measurable in prepubertal girls and markedly increase at puberty. The several-fold differential between prepubertal and pubertal-stimulated LH levels provides a reasonable discrimination. A peak stimulated serum LH level >5 IU/L alone is considered adequate evidence of HPG axis activation and hormonal maturation (maturing gonadotropin secretion) following previously established criteria (26–29).

### Psychological Scales

The Hamilton Anxiety Rating Scale (HAMA) was administered to evaluate anxiety symptoms on the day of MRI scanning. All children were also administered the complete C-WISC to screen for low intelligence prior to MR scanning. The primary care-giving parent or guardian of each child completed the Child Behavior Checklist (CBCL) (30), which is a psychological test that assesses a number of behavioral and emotional characteristics.

### Structural Data Acquisition

The MRI examinations were performed on the 3-T GE HDxt scanner (General Electric, Milwaukee, Wisconsin, USA) with an eight-channel phase array head coil. High-resolution T_1_-weighted images were acquired with a volumetric three-dimensional fast spoiled gradient recall (FSPGR) sequence. The scan parameters were as follows: repetition time = 8.89 ms, echo time = 4.02 ms, flip angle = 15°, field of view = 24 cm, voxel size = 1×1×1 mm^3^, and 160 slices with no gap. T1- and T2-weighted images were inspected and screened for scan artifacts and gross brain abnormalities by two experienced neuroradiologists.

### Image Processing

#### Surface-Based Analysis

Construction of the cortical surface using structural MRI data was performed with the FreeSurfer package (version 5.30, http://surfer.nmr.mgh.harvard.edu/). This method has been validated against histological analysis on postmortem brains (31) and manual measurements (17) and has high test–retest reliability (32, 33). Computational advances in surface reconstruction (34, 35) are beneficial to its use (36). During preprocessing, gray/white matter boundaries and the pia mater were automatically delineated. The cortical thickness was defined as the difference between equivalent vertices lying between the gray/white matter interface and the pia mater (37) using both intensity and continuity information from the entire three-dimensional MR volume in the segmentation and deformation procedures. Following registration of all subjects’ cortical reconstructions to a common average surface and the interpolation steps, the surface maps are capable of detecting submillimeter differences between groups (37). A detailed description of this approach is provided in the supporting information **Supplementary Methods**.

### Statistical Analysis

Differences in demographic characteristics [e.g., age and body mass index (BMI)], hormone concentrations, and psychological scale scores between the CPP and NCPP girls were examined using independent sample T-tests. Statistical significance was set at an alpha <0.05.

Vertex-wise comparisons of cortical maps between the two groups were conducted using a general linear model with age, weight and height as covariates. Because all images were aligned to a common template, we did not use the intracranial volume as a covariate (38). Since IQ has already been shown to influence cortical thickness (39), we ran an additional analysis including IQ as a covariate. A detailed description of this process is provided in the supporting information **Supplementary Analysis**. Prior to this process, a smoothing step with a 10-mm full width at half maximum was initiated to average the cortical thickness and volume data across participants in a common spherical coordinate system. Nonparametric cluster-wise corrections for multiple comparisons were performed using the FreeSurfer false discovery rate (FDR) or Monte Carlo simulation tool with a corrected cluster-forming threshold of *p* < 0.05 (18).

The average cortical thickness data were extracted from clusters showing significant differences between the groups. Associations between regional cortical parameters and the stimulated hormone levels were examined using regression analyses. To investigate the effect of anatomical maturation on psychosocial development, partial correlations were also conducted (corrected for age) between the cortical values and psychological features, including intellectual abilities and behavioral and emotional characteristics. We performed partial correlations (corrected for age) for the psychological scores and hormone levels, which showed significant correlations with the regional cortical parameters.

## Results

### Descriptive Statistics

No significant differences were found in age, weight, height, and BMI between the CPP and NCPP girls. The CPP girls showed higher stimulated plasma LH and E2 levels than the NCPP girls (**Table 1**). No significant differences between groups were found in the FSH, TES, PRL, and COR levels or the psychosocial features (*p* > 0.05; see supporting information in **Supplementary Table 1** for group comparisons of the C-WISC and CBCL subscales).

### Group Differences in Cortical Thickness

**Figure 1:**
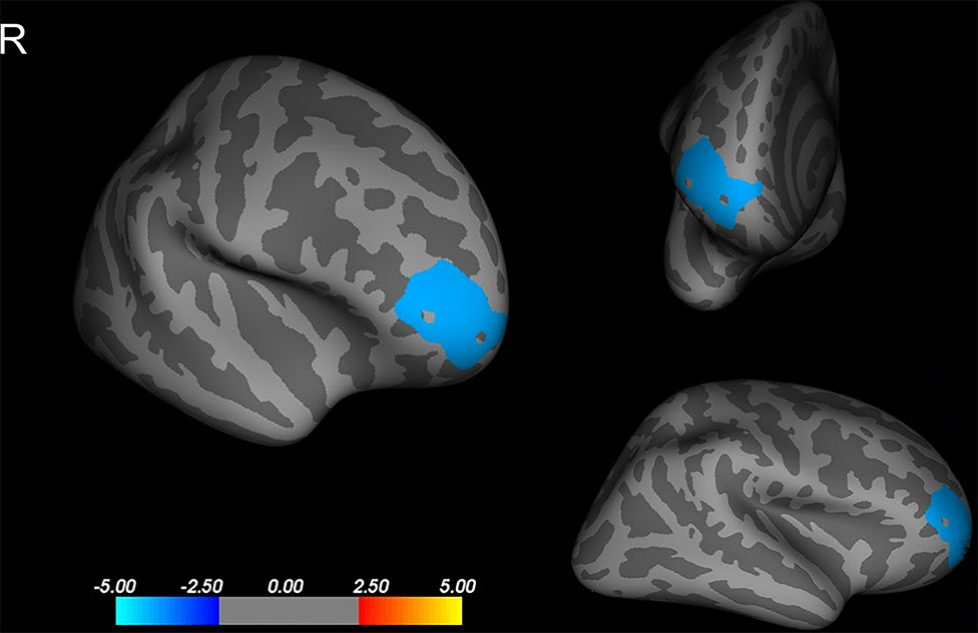
Differences in cortical thickness between girls with and without central precocious puberty. A thinner cortical thickness in the right rostral middle frontal cortex in the central precocious puberty (CPP) girls that co-varied by age, weight, and height is indicated by a blue/cool color (Monte Carlo simulation *p* < 0.05). R, right hemisphere.

Compared to that of the NCPP girls, the CPP girls showed less cortical thickness in the right rostral middle frontal cortex (*p* < 0.05, corrected using Monte Carlo simulation with 10,000 iterations; **Figure 1**). No significant difference between the groups was found in the left hemisphere (*p* > 0.05, corrected using FDR or Monte Carlo simulation).

### Associations Between Regional Cortical Parameters, Stimulated Hormone Levels, and Psychological Features

**Figure 2:**
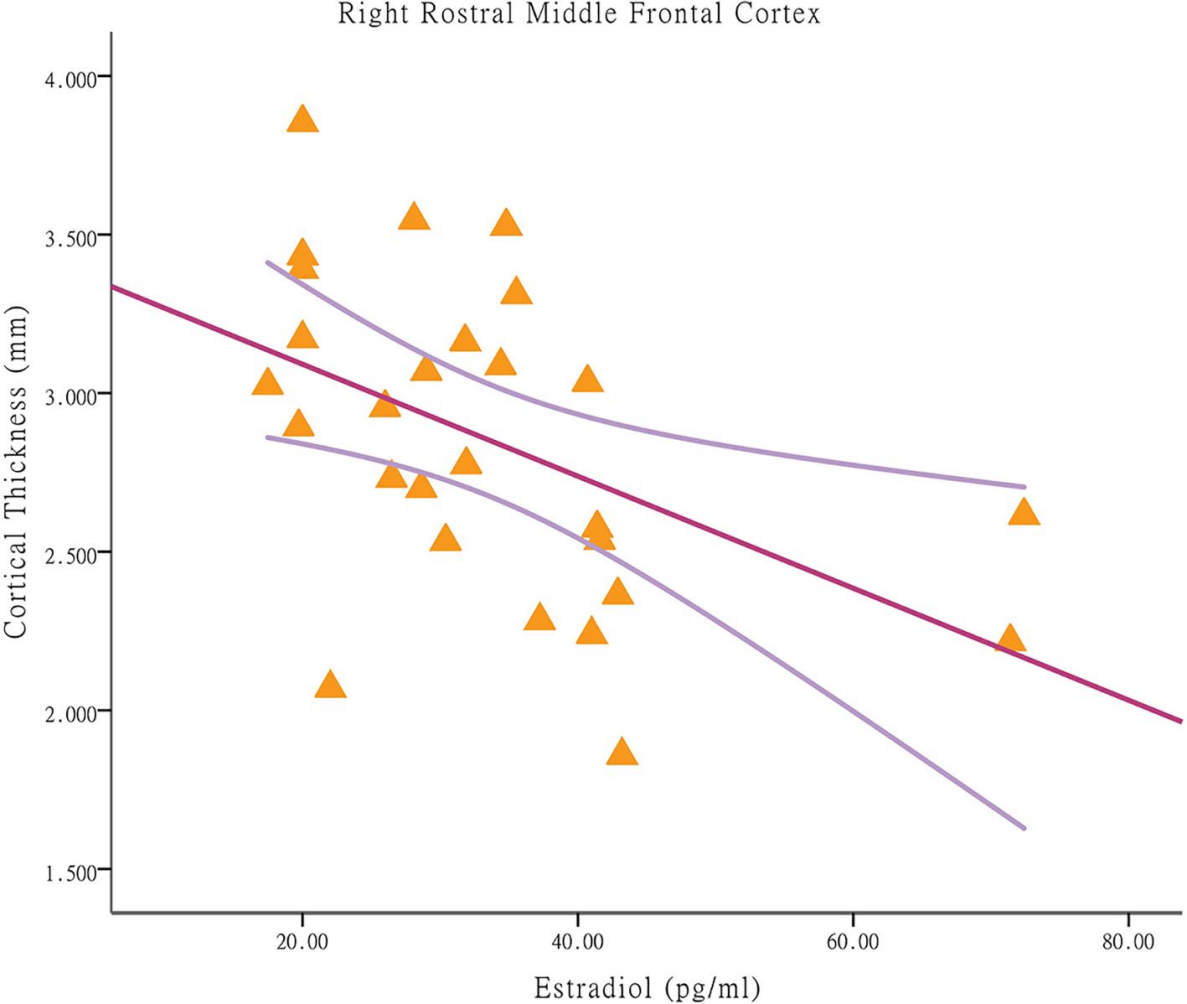
Associations between cortical thickness and stimulated estradiol concentrations in brain regions with significant between-group differences in central precocious puberty girls. Among central precocious puberty girls, less cortical thickness in the right rostral middle frontal cortex is associated with higher estradiol concentrations (*p* = 0.010, r = −0.485).

Among the CPP girls, less cortical thickness in the right rostral middle frontal cortex was associated with higher E2 concentrations [*p* = 0.010, r = −0.485; applying a Bonferroni correction at *p* < 0.025 (0.05/2); **Figure 2**]. The significant regression modal is as follows: right rostral middle frontal cortex thickness = 3.44-0.018E2 (*p* = 0.010). With respect to the partial correlation analysis, higher E2 concentrations were positively correlated with the hyperactivity score [*p* = 0.000, r = 0.775; Bonferroni correction at *p* < 0.004 (0.05/13)], which is a prominent behavioral characteristic of CBCL (after removal of one outlier). Among the NCPP girls, no significant correlations were found between the cortical thickness in regions with group differences and the stimulated hormone levels and psychological scores.

## Discussion

To the best of our knowledge, this study is the first to investigate the impact of premature HPG axis activation on cortical morphometry and its associations with cognitive and behavioral development in girls undergoing CPP defined by GnRH stimulation testing. Rather than finding widespread differences in brain anatomy between the CPP and NCPP girls, cortical thickness differences were evident specifically in the prefrontal cortex regions. The regression analysis revealed significant associations between the E2 levels and the cortical thickness in regions within the anterior middle frontal lobe. These findings suggest that the early hormonal changes induced by the onset of CPP are regionally specific to or more prominent in regions in the frontal lobe, which is known to be a cortical region with prolonged maturation during adolescence (40). Our findings of hormonal relationships with the neocortical structure in CPP girls suggest that the neurodevelopmental pattern of this neocortical region, which is known to be important for higher cognition and behavioral modulation, may be influenced by hormone-related changes during PP. Taken together, these results may facilitate the understanding of the role of CPP in several psychiatric disorders associated with prefrontal dysfunctions and behavior deficits occurring in adolescence.

Although the exact neurobiological processes underlying CPP in girls cannot be determined, specific cortical thickness differences in the prefrontal cortex were apparent. The most prominent cortical thinning was observed in the right rostral middle frontal cortex. This region is part of Brodmann area 10, which plays an important role in managing many executive functions, such as working memory and response inhibition (41), and is one of the least well understood regions of the human brain despite its involvement in mentalizing, personality expression, decision-making, and moderating social behavior (42, 43). In fact, previous studies of the cortical thickness or volume during normal pubertal development and in many teenage neuropsychiatric disorders also found positive results in the prefrontal lobe (44, 45). The cortical thickness and volume collectively confirmed the vulnerability of the prefrontal cortex (46). Since the cortical thickness can provide more details reflecting the size, density, and arrangement of neurons, neuroglia, and nerve fibers in the cortical columns (47, 48), our findings of changes in the cortical thickness in CPP girls can be posited to reflect early pubertal effects on these synaptic, dendritic, and axonal developmental processes. The thinning cortical thickness observed here may represent early modifications, such as cell death and neurite pruning (49). In fact, synaptic pruning in the cerebral cortex, including the prefrontal areas, generally is accepted to occur at puberty. Overproduction and developmental remodeling, including substantial elimination of synaptic spines, continue beyond adolescence until they stabilize at the adult level (50). However, determining whether any inverse influences underlie premature selective elimination of the initially overproduced synapses in CPP girls needs further investigation.

Another interesting finding was that a thinner cortical thickness of the right prefrontal lobe was positively associated with elevated E2 levels as measured by the GnRH stimulation test. Since CPP results from premature activation of the HPG axis, elevation of gonadotropin may stimulate the production of sex steroids, which leads to the production of estrogens in girls. Our result suggests that the decrease in the cortical thickness during the early phase of CPP may be directly or indirectly mediated by this E2 production process. Hormone-related modification of the prefrontal region may represent the core pathophysiology during the early course of CPP. Indeed, both animal and human studies have found that steroid hormones****exert a profound influence on the structure and function of the nervous system (51, 52), and the prefrontal cortex and its neural circuitry have been speculated to be mediators of estrogen (52). All key estrogen receptors present throughout the body are also present in synapses of the prefrontal cortex (53). However, most studies have supported trophic effects of estrogen, inducing neuronal survival, spinogenesis and synaptogenesis (54), although many inverse influences on the cortex microstructure have also been reported (55, 56). The underlying hormone-related refinement mechanisms, such as triggering selective neuro-anatomical alterations and eliminating initially overproduced synapses, await further confirmation.

However, previous studies found certain links between the cortical structure and testosterone during early brain development (57), but no significant correlation was found in our research. This discrepancy may due to the early pubertal phase during which CPP girls have not shown significant changes in the testosterone concentration; indeed, no differences were found between the CPP and NCPP girls in our samples. Moreover, all the girls in this study were in the very early pubertal stage without menophania. Without the confounding factors of the menstrual cycle, we can obtain more stable and representative pubertal hormone concentrations.

The positive correlation between the E2 concentration and hyperactivity means that HPG axis activation does have some influence on behavioral development. Because CPP girls have significantly elevated E2 concentrations, they may have higher hyperactivity scores and appear to be vulnerable to relevant behavioral symptoms. In fact, previous studies have suggested that CPP is associated with an increased risk of development of certain cognitive and behavioral problems (11, 58). Early in 1985, a standardized behavioral assessment of 33 girls with CPP have already suggested that girls with CPP scored high on the externalizing scale and the aggressive and hyperactive scales (59). However, these symptoms can be directly or indirectly mediated by biological, psychological, social, and environmental variables. Therefore, the extent of the association with hormonal changes is unclear. Studies in animals have supported the influence of estradiol on central nervous system differentiation and observed behavior, although the precise human behavioral and psychobiological effects have not been elucidated (60). Our findings may increase understanding of the endocrine mechanism underlying the developmental psychopathology.

Furthermore, previous studies have presented many cognitive and behavioral features that are closely associated with the frontal cortex (61). Previous neuroimaging investigations focused on separated cognitive elements found distinct frontal systems for response inhibition, attentional capture, and error processing (62). Animal studies in mice showed that an increase in ionotropic glutamate receptors activity in the prefrontal cortex contributed to some abnormal behaviors, such as hyperactivity (63). The hormone-related thinning of the prefrontal cortex found in our study might be a pathway to achieve hormone-related behavioral regulation. However, in our research, no significant correlation was found between the cortical thickness in regions with differences between groups and the psychological scores, and no differences in these scores were observed between the CPP and NCPP girls. These finding may have two explanations. First, the vertex-wise comparison of the cortical maps between the two groups has high sensitivity to detect small differences and can provide more detailed morphometric information, but these psychological scales only represent major changes in cognitive or behavioral development; thus, potential fine neuropsychological changes may not have been reflected on the corresponding scales. Second, since moving from a normal to an abnormal psychosocial state may require a conversion process, the psychological changes may not be as dramatic as the hormonal or morphological changes in early CPP girls. Additionally, some neurotransmitters may bridge the morphological and behavioral changes.

The current study has certain limitations. First, the present study was cross-sectional in nature and thus causality could not be asserted regarding whether the early phase of CPP caused brain structure alterations. Longitudinal human studies and experimental animal work are needed to establish causal effects. Second, the lack of measurement of the duration of hormone maturation is another potential limitation of this study. Third, other age-related factors (e.g., education and other environmental influences, such as social and emotional stresses) may impact behavioral correlations with our MRI and hormonal data. Fourth, healthy controls were not considered in this study because it is hard to convince the parents or guardians to let their normally developed girls to complete these exams (especially with the injection of medication). Finally, although our data show significant effects in girls with early CPP, whether similar hormone–neuroanatomy correlations are seen in girls at later stages after pubertal onset remains to be determined.

## Conclusions

Taken together, the results of the current study revealed specific changes in the prefrontal cortex that were related to hormonal changes in girls during premature HPG axis activation. This hormone-related modification may represent the core neurodevelopment pathophysiology during the early course of CPP, and it might be a pathway to achieve hormone-related behavioral regulation. These findings indicate an important role of hormonal axis activation in brain and psychosocial development during this sensitive developmental period.

## Ethics Statement

This study was carried out in accordance with the recommendations of “the medical ethics committee of the Second Affiliated Hospital and Yuying Children’s Hospital of Wenzhou Medical University” with written informed consent from all subjects. All subjects gave written informed consent in accordance with the Declaration of Helsinki. The protocol was approved by the “the medical ethics committee of the Second Affiliated Hospital and Yuying Children’s Hospital of Wenzhou Medical University.”

## Author Contributions

Drs DY, WZ and YZ conceptualized and designed the study, collected data, carried out the initial analyses, drafted the initial manuscript, and reviewed and revised the manuscript.

Prof PL conceptualized and designed the study, designed the data collection instruments, collected data, and reviewed and revised the manuscript critically for important intellectual content.

Drs BT, YF, YC, LZ, LL and XG carried out the further analyses, interpreted data, and reviewed and revised the manuscript critically for important intellectual content.

Profs XL, LR, JS and ZY conceptualized and designed the study, coordinated and supervised data collection, interpreted data, and reviewed and revised the manuscript critically for important intellectual content.

All authors approved the final manuscript as submitted and agree to be accountable for all aspects of the work.

## Funding

This work was supported by the National Natural Science Foundation of China (grant numbers 81371527, 81671664, and 81621003), the National Program for Special Support of Eminent Professionals, the National Program for Support of Top-notch Young Investigators, the Zhejiang Medical Health Science and Technology Program (grant number 2017KY108), the Zhejiang Provincial Natural Science Foundation (grant number LY19H180003), and the Medical Health Science and Technology Project of Zhejiang Province (grant number 2017ZD024).

## Conflict of Interest Statement

The authors declare that the research was conducted in the absence of any commercial or financial relationships that could be construed as a potential conflict of interest.

The handling Editor declared a shared affiliation, though no other collaboration, with several of the authors WZ, BT, LL, XG at the time of the review.
