# Supplementary material for: Initiation of the Hypothalamic–Pituitary–Gonadal Axis in Young Girls Undergoing Central Precocious Puberty Exerts Remodeling Effects on the Prefrontal Cortex
Source: Front Psychiatry. 2019 May 10;10:332. doi: 10.3389/fpsyt.2019.00332 (PMC6524415; doi:10.3389/fpsyt.2019.00332)
Supplement: Supplementary file 1 [file Table_1.docx]

***Supplementary Material***

1. **Supplementary Materials and Methods (SuppMethods)**

**1.1 Gonadotropin-releasing hormone (GnRH) stimulation test**

Following overnight fasting, the participants were asked to arrive at the hospital at approximately 8:00 am. Luteinizing hormone-releasing hormone (LHRH) was injected as an intravenous bolus of 2.5 μg/kg (maximum dose < 100 μg) ([Predieri et al., 2011](#_ENREF_7)) through an indwelling catheter. Four to five milliliters of blood were collected immediately before injection (0-minute sample), and then two milliliters were collected at 30 and 60 minutes after the injection.

The blood samples were sent for analysis to the hospital clinical laboratory (the 0-minute sample was delivered immediately, and the 30-minute sample was sent with the 60-minute sample). The samples were centrifuged, separated, and assayed. For the 0-minute sample, we assayed the LH, FSH, estradiol (E2), testosterone (TES), prolactin (PRL), and cortisol (COR) concentrations. For the 30-minute sample, we assayed the LH, FSH and E2 concentrations. For the 60-minute sample, we assayed the LH and FSH concentrations. Thus, the E2, LH and FSH concentrations had multiple time points, whereas the TES, PRL and COR concentrations were obtained only at baseline.

**1.2 Surface-based analysis**

The cortical surfaces were constructed based on structural MRI data with the FreeSurfer package (version 5.30, <http://surfer.nmr.mgh.harvard.edu/>). Computational advances in surface reconstruction ([Dale et al., 1999](#_ENREF_2); [Fischl et al., 1999](#_ENREF_5)) are an advantage to its use ([Winkler et al., 2010](#_ENREF_9)). During preprocessing, gray/white matter boundaries and the pia mater were automatically delineated. The cortical thickness was defined as the difference between equivalent vertices lying between the gray/white matter interface and the pia mater ([Fischl and Dale, 2000](#_ENREF_3)) using both intensity and continuity information from the entire three-dimensional MR volume during the segmentation and deformation procedures. Briefly, the main process of the surface-based pipeline included automated registration to the Talairach space, stripping the skull from the image sets, segmentation of the subcortical white matter and gray matter structures, intensity normalization, tessellation of the gray matter and white matter boundaries, automated topology correction, surface refinement and nudge following the intensity gradients to optimally place the gray/white and gray/CSF borders defined at the location with the greatest shift in signal intensity ([Dale et al., 1999](#_ENREF_2); [Fischl et al., 1999](#_ENREF_5); [Fischl and Dale, 2000](#_ENREF_3); [Fischl et al., 2002](#_ENREF_4); [Fischl et al., 2004](#_ENREF_6); [Segonne et al., 2004](#_ENREF_8)). Following registration of all subjects’ cortical reconstructions to a common average surface and the interpolation steps, the surface maps are capable of detecting submillimeter differences between groups ([Fischl and Dale, 2000](#_ENREF_3)).

**References**

Bajaj, S., Raikes, A., Smith, R., Dailey, N.S., Alkozei, A., Vanuk, J.R., et al. (2018). The Relationship Between General Intelligence and Cortical Structure in Healthy Individuals. *Neuroscience* 388**,** 36-44. doi: 10.1016/j.neuroscience.2018.07.008.

Dale, A.M., Fischl, B., and Sereno, M.I. (1999). Cortical surface-based analysis. I. Segmentation and surface reconstruction. *Neuroimage* 9(2)**,** 179-194. doi: 10.1006/nimg.1998.0395.

Fischl, B., and Dale, A.M. (2000). Measuring the thickness of the human cerebral cortex from magnetic resonance images. *Proc Natl Acad Sci U S A* 97(20)**,** 11050-11055. doi: 10.1073/pnas.200033797.

Fischl, B., Salat, D.H., Busa, E., Albert, M., Dieterich, M., Haselgrove, C., et al. (2002). Whole brain segmentation: automated labeling of neuroanatomical structures in the human brain. *Neuron* 33(3)**,** 341-355.

Fischl, B., Sereno, M.I., and Dale, A.M. (1999). Cortical surface-based analysis. II: Inflation, flattening, and a surface-based coordinate system. *Neuroimage* 9(2)**,** 195-207. doi: 10.1006/nimg.1998.0396.

Fischl, B., van der Kouwe, A., Destrieux, C., Halgren, E., Segonne, F., Salat, D.H., et al. (2004). Automatically parcellating the human cerebral cortex. *Cereb Cortex* 14(1)**,** 11-22.

Predieri, B., Luisi, S., Casarosa, E., Farinelli, E., Antoniazzi, F., Wasniewska, M., et al. (2011). Allopregnanolone levels decrease after gonadotropin-releasing hormone analog stimulation test in girls with central precocious puberty. *Journal of Endocrinological Investigation* 34(1)**,** 38-44. doi: 10.3275/7100.

Segonne, F., Dale, A.M., Busa, E., Glessner, M., Salat, D., Hahn, H.K., et al. (2004). A hybrid approach to the skull stripping problem in MRI. *Neuroimage* 22(3)**,** 1060-1075. doi: 10.1016/j.neuroimage.2004.03.032.

Winkler, A.M., Kochunov, P., Blangero, J., Almasy, L., Zilles, K., Fox, P.T., et al. (2010). Cortical thickness or grey matter volume? The importance of selecting the phenotype for imaging genetics studies. *Neuroimage* 53(3)**,** 1135-1146. doi: 10.1016/j.neuroimage.2009.12.028.

1. **Supplementary Table**

**Supplementary Table (SuppTable) 1.** The 2-tailed t tests comparing the intelligence quotient and child behavior checklist data from the CPP and NCPP girls

| **Characteristic** | | **CPP (n=28)** | | | **NCPP (n=37)** | | ***P*** |  |
| --- | --- | --- | --- | --- | --- | --- | --- | --- |
|  |  | **Mean** | | **SD** | **Mean** | **SD** |  |  |
| **IQ** |  | |  |  |  |  |  |  |
| Information | | | 6.09 | 2.07 | 7.81 | 3.02 | 0.103 |  |
| Similarities | | | 8.64 | 3.20 | 10.76 | 3.45 | 0.100 |  |
| Arithmetic | | | 6.73 | 2.61 | 7.67 | 3.35 | 0.426 |  |
| Vocabulary | | | 7.45 | 2.58 | 8.90 | 3.49 | 0.235 |  |
| Comprehension | | | 4.91 | 2.80 | 7.05 | 4.01 | 0.127 |  |
| Picture completion | | | 6.27 | 1.34 | 6.57 | 1.69 | 0.616 |  |
| Picture arrangement | | | 8.09 | 2.42 | 8.38 | 1.32 | 0.718 |  |
| Block design | | | 10.18 | 3.31 | 10.10 | 2.91 | 0.940 |  |
| Object assembly | | | 9.18 | 3.68 | 10.14 | 4.35 | 0.538 |  |
| Coding | | | 10.73 | 4.29 | 12.71 | 2.91 | 0.188 |  |
| VIQ | | | 78.18 | 12.84 | 89.33 | 16.44 | 0.060 |  |
| PIQ | | | 91.36 | 14.97 | 96.14 | 13.17 | 0.359 |  |
| IQ (FIQ) | | | 82.82 | 14.07 | 92.05 | 13.98 | 0.087 |  |
| **CBCL** | | |  |  |  |  |  |  |
| Activities | | | 6.52 | 2.74 | 6.53 | 2.46 | 0.989 |  |
| Social Skills | | | 7.41 | 2.03 | 7.37 | 2.53 | 0.934 |  |
| Learning Ability | | | 4.74 | 1.02 | 4.38 | 1.24 | 0.227 |  |
| I Depression | | | 1.89 | 2.24 | 1.75 | 1.93 | 0.793 |  |
| II Social Withdrawal | | | 1.37 | 1.86 | 0.72 | 1.00 | 0.110 |  |
| IIISomatic Complaints | | | 0.63 | 0.63 | 1.19 | 1.70 | 0.074 |  |
| IV Schizoid | | | 0.11 | 0.32 | 0.28 | 0.57 | 0.145 |  |
| V Hyperactivity | | | 1.00 | 1.41 | 1.17 | 1.52 | 0.659 |  |
| VI Sexual Problems | | | 0.48 | 0.94 | 0.56 | 0.88 | 0.748 |  |
| VII Delinquent Behavior | | | 0.11 | 0.32 | 0.18 | 0.46 | 0.532 |  |
| VIII Aggressive Behavior | | | 2.30 | 3.04 | 2.22 | 2.57 | 0.917 |  |
| IX Cruelty | | | 0.22 | 0.42 | 0.18 | 0.58 | 0.731 |  |
| Total Score | | | 7.70 | 8.00 | 9.39 | 9.14 | 0.448 |  |

Abbreviations: SD: standard deviation; IQ: Intelligence Quotient; VIQ: Verbal Intelligence Quotient; PIQ: Performance Intelligence Quotient; FIQ: Full-Scale Intelligence Quotient; CBCL: Child Behavior Checklist.

1. **Supplementary Analysis**

**Since IQ has already been shown to influence cortical thickness(**[**Bajaj et al., 2018**](#_ENREF_1)**), and there is a 10-point difference in mean total IQ scores in the two groups and this nearly achieves significance (p=0.09 overall and 0.06 for verbal IQ).We have run an additional analysis including IQ as a covariate to rule out any possibility that it is affecting our findings.**

**The results were consistent with the previous findings(Compared to that of the NCPP girls, the CPP girls showed less cortical thickness in the right rostral middle frontal cortex (p <0.05, corrected using Monte Carlo simulation with 10,000 iterations)).**
